# From Traditional Use to Molecular Mechanisms: A Bioinformatic and Pharmacological Review of the Genus *Kalanchoe* with In Silico Evidence

**DOI:** 10.3390/biotech14040097

**Published:** 2025-12-12

**Authors:** Cristián Raziel Delgado-González, Ashutosh Sharma, Margarita Islas-Pelcastre, Mariana Saucedo-García, Eliazar Aquino-Torres, Jaime Pacheco-Trejo, Silvia Armenta-Jaime, Nallely Rivero-Pérez, Alfredo Madariaga-Navarrete

**Affiliations:** 1 Área Académica de Ciencias Agrícolas y Forestales, Instituto de Ciencias Agropecuarias, Universidad Autónoma del Estado de Hidalgo, Tulancingo 43600, Hidalgo, Mexico; cristian_delgado@uaeh.edu.mx (C.R.D.-G.); mislas@uaeh.edu.mx (M.I.-P.); saucedo@uaeh.edu.mx (M.S.-G.); eaquino@uaeh.edu.mx (E.A.-T.); jaime_pacheco@uaeh.edu.mx (J.P.-T.); silvia_armenta@uaeh.edu.mx (S.A.-J.); 2 School of Engineering and Sciences, Centre of Bioengineering, Tecnológico de Monterrey, Av. Epigmenio González No. 500, Fracc. San Pablo, Querétaro 76130, Querétaro, Mexico; ashutosh.bt@gmail.com; 3 Área Académica de Medicina Veterinaria y Zootecnia, Instituto de Ciencias Agropecuarias, Universidad Autónoma del Estado de Hidalgo, Tulancingo 43600, Hidalgo, Mexico; nallely_rivero@uaeh.edu.mx

**Keywords:** pharmacological activity, bioinformatics, secondary metabolism, molecular docking

## Abstract

The genus *Kalanchoe* (Crassulaceae) comprises approximately 125 species of succulents distributed across Madagascar, Africa, Arabia, Australia, Southeast Asia, and tropical America. Traditionally regarded as “miracle plants”, *Kalanchoe* species are employed for treating inflammatory, infectious, metabolic, and cardiovascular conditions; this is associated with their abundant content of polyphenols, including phenolic acids and flavonoids such as quercetin, kaempferol, luteolin, rutin, and patuletin. However, robust clinical evidence remains limited. This review integrates pharmacological and bioinformatic perspectives by analyzing more than 70 studies published since 2000 on 15 species, including *Bryophyllum*. As an in silico complement, the genome of *Kalanchoe fedtschenkoi* was used to predict genes (AUGUSTUS), perform homology searches against *Arabidopsis thaliana*, and model three key enzymes: CHS, CYP90, and VEP1. The AlphaFold2/ColabFold models showed conserved catalytic motifs, and molecular docking with representative ligands supported the plausibility of biosynthetic pathways for flavonoids, brassinosteroids, and bufadienolides. The available evidence highlights chemopreventive, antibacterial, anti-inflammatory, antiviral, antioxidant, and cytotoxic activities, primarily associated with flavonoids and bufadienolides. Significant gaps remain, such as the lack of gene–metabolite correlations and the absence of standardized clinical trials. Overall, *Kalanchoe* represents a promising model that requires multi-omics approaches to enhance its phytopharmaceutical potential.

## 1. Introduction

The *Kalanchoe* genus belongs to the Crassulaceae family and comprises succulent perennial plants characterized by their fleshy leaves, high adaptability, and notable capacity for vegetative reproduction [1]. The genus includes approximately 125 recognized species, among which *Kalanchoe brasiliensis* (Cambess) and *Kalanchoe pinnata* stand out due to their richness in polyphenolic compounds, including flavonoids and phenolic acids such as p-hydroxycinnamic, caffeic, p-coumaric, ferulic, p-hydroxybenzoic, protocatechuic acids, quercetin, kaempferol, luteolin, astragalin, rutin, and patuletin [2]. These phytochemical features have supported the growing interest in their biological and pharmacological potential.

Species of the Kalanchoe genus are widely distributed across tropical and subtropical regions of the world, particularly in Madagascar, southern and eastern Africa, Arabia, Australia, Southeast Asia, and tropical America, including Panama, Costa Rica, Colombia, Venezuela, Mexico, and Nicaragua [3]. Their ecological plasticity has facilitated both natural and anthropogenic dissemination.

*Kalanchoe* spp. possess numerous registered ethnobotanical uses [4]; in several regions, they are commonly referred to as “miracle leaf” due to their traditional application in the treatment of diverse ailments [5], notably their recognized anti-inflammatory properties [6]. For example, extracts from K. *daigremontiana* exhibit cytotoxic, antimicrobial, antioxidant, and sedative activities, among others [7], supporting their recognition as medicinally valuable plants. Beyond in vitro findings, *Kalanchoe* leaves are employed as anti-inflammatory and antiseptic remedies [8], as well as in the management of cardiovascular disorders [9], diabetes, and various tumoral processes [10,11]. These medicinal applications are generally attributed to high concentrations of bioactive compounds such as flavonols and phenolic acid glycosides [12], particularly quercetin derivatives; however, clinical validation of these effects remains limited [13].

From an ecological perspective, several *Kalanchoe* species are classified as invasive. In certain environments, they can transform forests or savannas into grasslands and may facilitate the proliferation of other invasive organisms, contributing to ecological “invasive meltdown” [14]. The coexistence of exotic and native species can reduce local genetic diversity, thus affecting ecosystem stability [15,16].

In recent years, advances in biotechnology have begun to shed light on aspects of *Kalanchoe* biology relevant to its medicinal potential, including transcriptomic analyses, biosynthetic pathway elucidation, and in silico approaches aimed at identifying genes associated with the production of bioactive metabolites. These developments offer tools to better understand the molecular basis of their therapeutic properties and to support future pharmacological applications.

Although *Kalanchoe* metabolites, particularly bufadienolides and flavonoids, show promising bioactivities, the development of validated therapeutic products remains limited. No *Kalanchoe*-derived compounds have been approved for clinical use, and current commercial formulations are restricted to non-standardized traditional herbal preparations lacking rigorous phytochemical and clinical validation.

The objective of this review is to provide updated information on the biological activity of the Kalanchoe genus and to integrate available in silico evidence, offering new perspectives that should be considered for guiding future research on this subject.

## 2. Materials and Methods

Over 70 articles containing clear evidence on the biological and pharmacological importance of *Kalanchoe* species, published from 2000 onwards, were reviewed. Articles with no substantial evidence, no biological/pharmacological activity, or unclear methodology were excluded from this study. There is taxonomic ambiguity between *Kalanchoe* and *Bryophyllum* and they were previously considered two different genera; another proposal has divided the genus *Kalanchoe,* positioning *Bryophyllum* as a subgenus or section of it. For this reason, the latter was also considered in the review. The investigation included 15 species of *Kalanchoe* and the *Bryophyllum* section to compare the pharmacological evidence and the patents associated with the species and their biological importance. The main academic search engines used were ResearchGate, PubMed, BASE, and Science Direct. As a section of the *Kalanchoe* genus, *Bryophyllum* species were also included in the review to include more studies related to the *Kalanchoe* genus.

To conduct a complementary exploratory analysis using bioinformatics tools, the sequenced genome of *Kalanchoe fedtschenkoi* was used as a model.

The corresponding sequences were obtained from the assembled GCA_002312845.1_K_fedtschenkoi_M2_v1 (National Center for Biotechnology Information (NCBI), Bethesda, MD, USA) genome and its genomic annotation [17,18]. The genes *g1020* (CHS), *g1408.t1* (CYP90), and *g827.t1* (VEP1) were included, as they are the most representative genes related to the bioactive products compared in this literature review.

Protein prediction was performed by processing contigs > 10 kb using AUGUSTUS (v3.4.0, University of Greifswald, Greifswald, Germany), dividing the genome into 200 kb blocks. The result was a set of 1281 predicted proteins [19].

A reference database of key *Arabidopsis thaliana* proteins (CHS, CYP90A1, VEP1/5β-reductase) was generated. A similarity search was performed using BLASTp (v2.12.0, National Center for Biotechnology Information (NCBI), Bethesda, MD, USA), with an E-value threshold ≤ 1 × 10^−5^ [20]. The main findings were as follows: CHS (g1020.t1): 81.9% identity, E-value = 0.0; CYP90 (g1408.t1): 40% identity, E-value = 1.6 × 10^−9^; and VEP1 (g827.t1): 53.6% identity, E-value = 2.9 × 10^−18^.

The three candidate sequences underwent structural prediction using ColabFold (v1.5.2, European Molecular Biology Laboratory (EMBL), Heidelberg, Germany; and University of Tokyo, Tokyo, Japan) (AlphaFold2) with MMseqs2 (v17, Max Planck Institute for Biophysical Chemistry, Göttingen, Germany) for multiple sequence alignment (MSA) [21].

Five models were generated per protein; rank-1 models were retained for further analysis [21,22]. The pLDDT (local confidence) and PAE (Predicted Aligned Error) metrics were evaluated to estimate folding accuracy and domain–domain interactions. Catalytic and structural motifs were identified using regular expressions (regex) and visual confirmation: CHS (chalcone synthase): typical Cys–His–Asn catalytic triad of type III polyketide synthases (PKS). CYP90 (cytochrome P450): EXXR (K helix), PERF (meander), and FxxGxxxCxG (axial cysteine coordinating heme Fe) motifs. VEP1 (5β-reductase): TGxxxGIG/TGWxxGIG (NADPH cofactor binding) motifs and Tyr–Lys (YxxxK) catalytic pair. Models were analyzed in PyMOL (v2.5, Schrödinger LLC, New York, NY, USA), stained by pLDDT (50–100) [23]. In CYP90, a heme group was introduced by overlaying with P450cam (PDB 2CPP) to locate the catalytic site. In CHS, the model was aligned with the crystallographic enzyme from Medicago sativa (PDB 1CGK). In VEP1, the Tyr–Lys pair was defined as the catalytic center.

The receptors were generated in .pdbqt format using Open Babel (v3.1.1, The Open Babel Project, USA) and Meeko (v0.4.0, Meeko Scripps Research, San Diego, CA, USA). The ligands were optimized in 3D with Gasteiger loading [24,25] as follows: for CHS, p-coumaroyl-CoA, malonyl-CoA, and naringenin chalcone were used; for CYP90, campestanol (steroidal substrate) was used; and finally, for VEP1, progesterone was used as a ligand.

The dockings were performed using AutoDock Vina (v1.2.3, Scripps Research Institute, San Diego, CA, USA) with boxes centered on the catalytic residues of each model, with exhaustiveness values between 8 and 24 [26].

Two-dimensional protein–ligand interaction diagrams were generated from the molecular docking results obtained with AutoDock Vina (v1.2.3, Scripps Research Institute, San Diego, CA, USA) [26] using the .pdbqt output files corresponding to the CHS, CYP90, and VEP1 (POR) proteins and their respective ligands: malonyl-CoA, p-coumaroyl-CoA, naringenin chalcone, campestanol, quercetin, and progesterone.

To visualize the interactions, a Python (v3.10, Python Software Foundation, Wilmington, DE, USA) environment [27] under WSL-Ubuntu (v22.04, Canonical Ltd., London, UK) [28] was used, configured with the Matplotlib (v3.7.2, Matplotlib Development Team, USA) [29] and Pandas (v2.1.4, Pandas Development Team, USA) [30] libraries, in a reproducible analysis environment. The receptor and ligand .pdbqt files were processed using custom Python scripts to extract atomic coordinates and calculate interaction distances (cutoffs of 3.5 Å for hydrogen bonds and 4.2 Å for van der Waals contacts). The graphic style was based on the conventions of LigPlot+ (v2.3, European Bioinformatics Institute (EMBL-EBI), Hinxton, UK) [31] and Discovery Studio Visualizer (v2025, Dassault Systèmes BIOVIA, San Diego, CA, USA) [32], with modifications to maintain visual consistency among the analyzed proteins.

## 3. Results

Fifteen species of the *Kalanchoe* genus, including the *Bryophyllum* section, were reviewed in terms of studies related to their biological activity. *K. pinnata* was the most abundant species (Table 1). The activities associated with *K. pinnata* are hepatoprotective, antileishmanial, anti-tumor, nephroprotective, antioxidant, insecticidal, antiviral, antihypertensive, antidiabetic, and anti-inflammatory activities, among many others. As reported in Table 1, the activities reported for the most studied species, *K. pinnata*, are shared with other species of the same genera.

**Figure 1:**
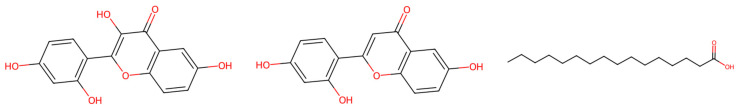
Representative chemical structures of natural compounds identified in *Kalanchoe* species: quercetin (**left**), kaempferol (**center**), and palmitic acid (**right**).

Many studies do not report the exact bioactive compound studied, only referring to different types of extracts; however, the most reported compounds among the *Kalanchoe* species are kaempferol and derivates, bufadienolides, bryophilline A, B, and C, quercetin, and other organic acids, such as palmitic acid (Figure 1).

All of these studies have mainly been conducted in the laboratory; however, clinical trials are an important step in the development of specific treatment for diverse diseases and ailments.

### 3.1. Clinical Trials

Six clinical trials registered in the U.S. National Library of Medicine database [63] have been reported for *Bryophyllum* species, including for treating conditions such as anxiety, sleep disorder, nocturia, overactive bladder, incontinence, tocolysis, labor-related conditions, preterm labor, and sleep quality in pregnancy. Two out of the six trials of *Bryophyllum* have been completed, one is terminated, one has not yet recruited people for the trial, one is actively recruiting participants, and the last one’s status is unknown. No clinical trials have been registered specifically under the *Kalanchoe* genus. However, only three articles have been published: “Two Randomised Clinical Trials on the Use of *Bryophyllum pinnatum* in Preterm Labour: Results after Early Discontinuation” [64], “Sleep quality in pregnancy during treatment with *Bryophyllum pinnatum*: an observational study” [65], and “Randomized, double-blind placebo-controlled trial with *Bryophyllum pinnatum* versus placebo for the treatment of overactive bladder in postmenopausal women” [66]. Analyzing the data and comparing them with all of the properties reported for the *Kalanchoe* genus, only a small fraction of the reported biological activities are mentioned in the published clinical trials. Of the six clinical trials identified, five were conducted in Switzerland, mainly at institutions such as University Hospital Zurich and affiliated gynecology departments (Table 2).

### 3.2. Product List/Patent

A total of 104 patents related to *Kalanchoe* species and the *Bryophyllum* section have been registered, including biotechnological, pharmacological, breeding, taxonomic, extract composition, and other applications. However, since the purpose of this review is mainly focused on the biological activity of the genus’ compounds, Table 3 only presents 16 registered patents related to its pharmacological or biological activity overall.

The most common patents are related to antioxidant, skin care and protection, cosmetic, and dermatological activities; just one patent is related to the treatment of prostate cancer.

Of the 16 patents presented in Table 3, none are related to currently registered clinical trials (Table 2).

Despite multiple patents being granted for Kalanchoe-derived products, translation into clinical development remains limited due to key barriers, including insufficient pharmacokinetic and toxicological profiling of major metabolites (e.g., bufadienolides), lack of standardized extraction and quality control procedures, regulatory constraints for complex botanical mixtures, and limited preclinical mechanistic evidence to justify progression to human trials.

### 3.3. Bioinformatics Analysis

We evaluated the predicted structure of type III polyketide synthase (PKS) based on its expected architecture and conserved catalytic features. Type III PKSs typically adopt a β-trefoil fold with a central active-site cavity formed by catalytic residues that guide polyketide chain initiation and elongation. Therefore, we expected a compact monodomain structure with high local confidence in the catalytic core.

To further validate the AlphaFold predictions, each model was structurally aligned with the closest homologous experimentally resolved structure available in the Protein Data Bank. The overlays show excellent conservation of the canonical fold expected for each enzyme class (Supplementary Figures S1–S3). Because no crystallographic structure is available for VEP1, we aligned the AlphaFold model to 4JIR, a representative aldo-keto reductase (AKR) with a well-resolved canonical fold. VEP1 is predicted to belong to the AKR/SDR enzyme family based on its cofactor-binding loop, catalytic tyrosine–lysine pair, and overall domain architecture. The structural overlay confirms that the predicted VEP1 model adopts the expected AKR-like fold (Supplementary Figure S3).

**Figure 2:**
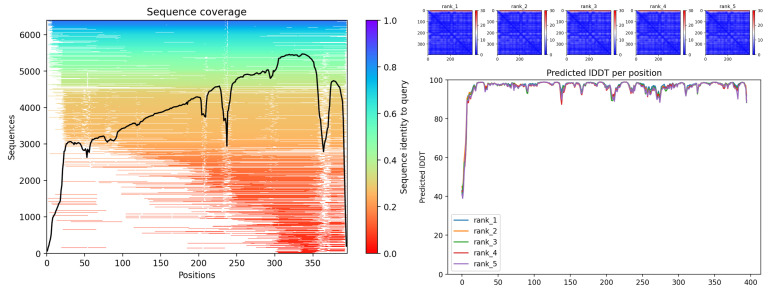
CHS (g1020). Sequence coverage of the MSA; black line indicates coverage by position (**left**); predominantly blue PAE maps (rank 1–5) indicate low error (**top right**) and pLDDT by residue with high confidence in the core and drops in the loops/terminal (**bottom right**).

The model showed high structural confidence (pLDDT > 80) and conservation of the β-trefoil fold typical of PKS. The model shows very high pLDDT (>90) throughout almost the entire protein, with localized dips in loops (~170, 220–240, and 350–380 aa) at the ends, consistent with mobile regions that modulate access to the catalytic pocket in type III PKS. The PAE maps are uniformly blue, consistent with a single, well-defined domain. The MSA coverage is broad, with local valleys coinciding with variable loops. The topology is consistent with a canonical CHS and supports its functional assignment (Figure 2).

**Figure 3:**
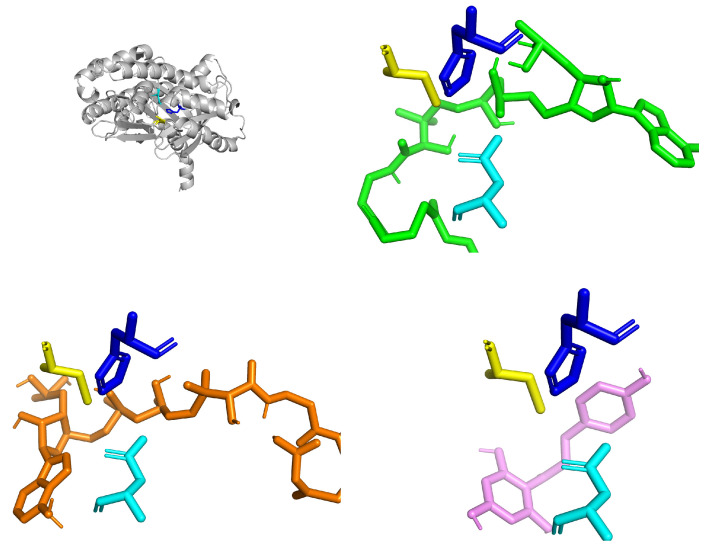
Predicted three-dimensional structure of the chalcone synthase (CHS) from *Kalanchoe fedtschenkoi*, represented as a ribbon (gray) showing the catalytic triad Cys164–His303–Asn336 highlighted in yellow, blue, and cyan, respectively. The image illustrates the localization of the active site in the center of the β-trefoyl domain characteristic of CHS (**top left**), molecular coupling of the substrate p-coumaroyl-CoA (green) to the active site of chalcone synthase (CHS) (**top right**), molecular coupling of the co-substrate malonyl-CoA (orange) to the catalytic site of CHS (**bottom left**), and binding of the naringenin chalcone product (pink) to the catalytic site of CHS (**bottom right**).

Alignment with the CHS of A. thaliana confirmed the Cys164–His303–Asn336 triad. The ligands corroborated the catalytic activity of the putative molecule. The ligands were naringenin chalcone (product, −8.3, greater stability, remains in the catalytic cavity), p-Coumaroyl-CoA (initial substrate, −7.2 at Cys164, position consistent with nucleophilic attack), and Malonyl-CoA (co-substrate, −6.8, oriented towards His303 with an elongating role). The catalytic cavity delimited by the triad (Cys in yellow, His in blue, Asn in cyan) showed a topology consistent with the Claisen condensation mechanism (Figure 3).

The model shows the thioester group positioned in a convergent orientation toward the catalytic triad (Cys164–His303–Asn336), consistent with its role as a malonyl unit donor during polytetracycline elongation. The p-coumaroyl-CoA ligand is oriented adjacent to the catalytic cysteine (Cys164), in close proximity to histidine (His303) and asparagine (Asn336), consistent with the CoA transfer mechanism in the first Claisen condensation. Meanwhile, the naringenin chalcone ligand is partially located in the hydrophobic cavity of the catalytic channel, oriented toward the active cysteine, suggesting an adjustment of the active site following cyclization and release of the final product.

With the CHS pocket, the three ligands establish a mixed network of hydrogen bonds and hydrophobic contacts. Docking analyses showed that the predicted ligand poses closely reproduced the canonical binding mode observed in the co-crystal structures 1CGK and 2CPP. In our simulations, the ligands consistently inserted into the active-site pocket along the same axis reported for chalcone intermediates in the crystal structures, with the aromatic rings positioned toward the entrance of the cavity and the electrophilic region oriented toward the catalytic Cys–His–Asn triad.

While small differences in rotational angle and burial depth were observed, these are consistent with the increased conformational freedom inherent to docking simulations. Overall, the docking results corroborate the experimentally validated ligand orientation characteristic of type III PKS enzymes.

**Figure 4:**
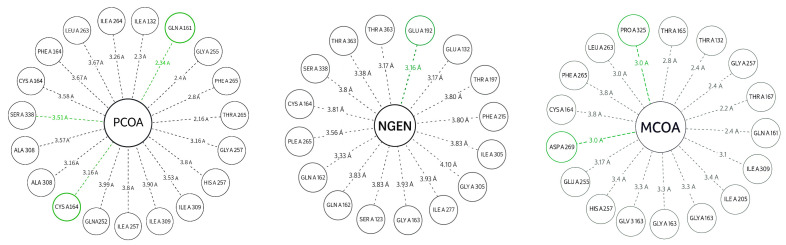
H bonds and hydrophobic contacts with residues of the catalytic pocket; the number in the lines allow us to appreciate which interactions are closer and, therefore, potentially more relevant for the three ligands: p-Coumaroyl-CoA (**left**), Malonyl-CoA (**center**) and Naringenin-chalcone (**right**), Green lines represent hydrogen bonds between the ligand and specific amino-acid residues, with distances falling within the defined cutoff for directional interactions, gray lines indicate van der Waals or hydrophobic contacts, illustrating non-specific proximal interactions between the ligand and surrounding residues.

MCOA (Figure 3) exhibits the highest total number of contacts due to its larger size, with several short H-bonds (≤3.0–3.2 Å) to polar residues in the vicinity of the active site and multiple vdW contacts along the coenzyme, suggesting multisite anchoring. PCOA (Figure 3) displays directed H-bonds in the CoA group region and a significant hydrophobic contribution from the aromatic ring, consistent with stable occupation of the hydrophobic channel. NGEN (Figure 4) exhibits a network of primarily hydrophobic interactions with some spot H-bonds; its planar geometry favors stacking and short contacts in the heart of the pocket. Vina’s affinities support the visual pattern: shorter contacts and thicker lines tend to correspond to more favorable positions.

**Figure 5:**
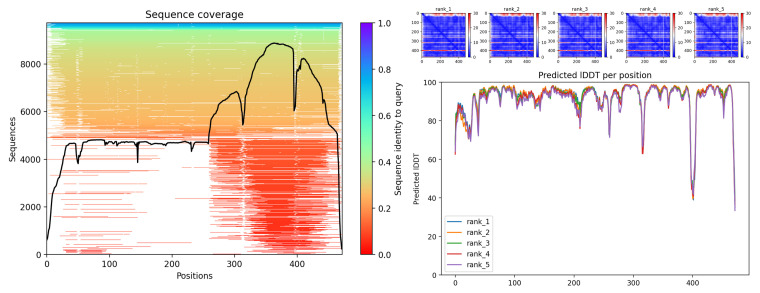
MSA sequence coverage; valleys in variable loops and extremes (**left**), PAE maps (rank 1–5) with low global error and warm bands at extremes (**top right**) and pLDDT by residue: high in the core, low in N-terminal/C-terminal and BC/FG loops (**bottom right**).

For the second putative protein, cytochrome P450 (CYP90; g1408.t1), pLDDT is high in the globular core (≈90–95 aa) with decreases in the N-terminal (≈1–15 aa), loops (≈170–190, 260–270, 390–410 aa), and C-terminal (~470–480 aa). PAE maps show low overall errors and warm bands at the ends, consistent with flexibility in the N-terminal transmembrane segment and C-terminal tail. MSA coverage is extensive, with valleys in BC/FG loops and near-meander/heme regions. These patterns, together with the primary homology (40% identity/440 aa; E = 1.6 × 10^−9^), strongly support the notation as CYP90-like (Figure 5).

**Figure 6:**
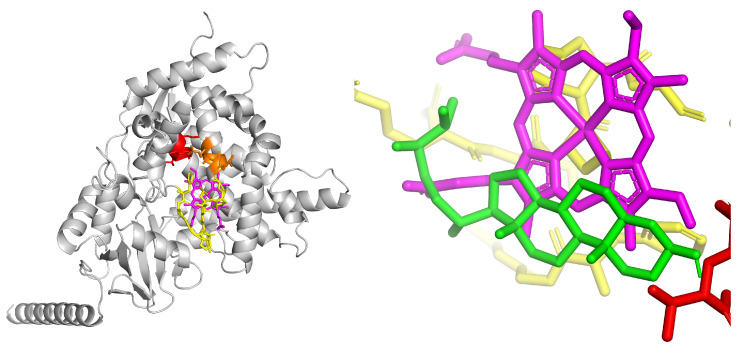
Active site and conserved motifs (**left**). Gray cartoon of the AlphaFold model (rank 1). Orange shows the EXXR motif and red the PERF motif; yellow shows the FxxGxxxCxG motif containing the conserved axial Cys; magenta shows the heme group transferred by superposition with P450cam (2CPP). The image is centered on the catalytic pocket, and a three-dimensional representation of the molecular docking of campestanol is shown (**right**).

The model predicted the typical folding of plant P450s, with well-defined EXXR, PERF, and FxxGxxxCxG motifs. Cys418 (yellow motif) coordinated the heme Fe (magenta) introduced by overlap with 2CPP (Figure 6). Coupling with campestanol yielded energies between −6.4 and −8.0 kcal/mol, highlighting an optimum mode at −8.0 kcal/mol.

The protein is shown in gray, the heme group in magenta, and the campestanol ligand in green. The catalytic pocket is delimited by the PERF, EXXR, and FxxGxxxCxG motifs, with the Cys418 residue coordinating to the central iron atom of the heme. The ligand was oriented toward the heme iron at a catalytically plausible distance (~3 Å), consistent with steroidal hydroxylation reactions typical of CYP90A1.

**Figure 7:**
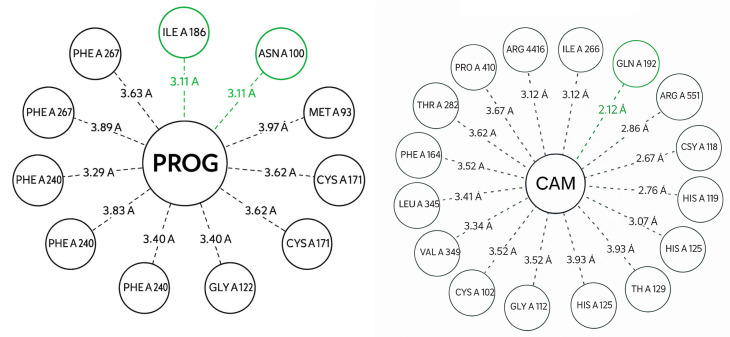
Two-dimensional diagram of interactions between CYP90 and CAM (**right**). The central node is the ligand abbreviation; circles represent residues in the pocket near the heme. Dashed green lines are hydrogen bonds (≤3.5 Å) and dashed gray lines are hydrophobic/van der Waals contacts (≤4.2 Å). A similar 2D diagram of interactions between VEP1 (POR) and PROG (progesterone) is shown (**left**).

Campestanol establishes close contacts (predominant vdW) with some point H-bonds in the vicinity of the channel; the pattern is consistent with a bulky steroid fitting to the pocket, and its log was used to annotate the affinity where interpretable. Figure 7 highlights which residues are closest (labeled circles) and the exact distance of the minimum contact. This allows for rapid identification of which positions within the CYP90 channel might be critical for substrate recognition and catalytic modulation.

**Figure 8:**
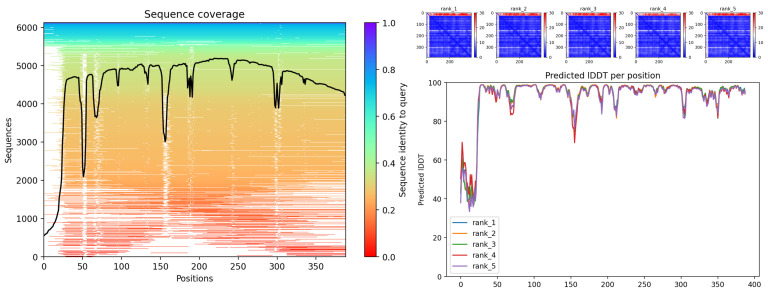
Sequence coverage of the MSA with local valleys in variable loops (**left**), PAE maps (rank 1–5) with low global error and higher uncertainty in the N-terminal (**top right**) and pLDDT per residue: high in the Rossmann-like core and low in the first ~25 aa (**bottom right**).

Finally, for 5β-reductase/VEP1 (g827.t1), the AlphaFold model revealed the SDR architecture with the Tyr381–Lys385 pair and the TGxxxGIG motif. The MSA shows good coverage along the core (≈30–350 aa) with localized steep dips (~50–60, ~150–160, and near ~300 aa), typical of variable loops. The PAE maps are mostly blue, with a warm band in the N-terminal (≈1–25 aa) suggesting flexibility of the end and possibly of the cofactor-binding segment. The pLDDT is low in the first ~25 aa and high in the Rossmann-like core; it exhibits additional discrete valleys (≈150–170, ~210, and ~340–360 aa) consistent with mobile loops. Overall, the model is consistent with an SDR/AKR-type enzyme. Since the primary homology support comes from a relatively short alignment (53.6% over 69 aa; E = 2.9 × 10^−18^), it is recommended to validate the integrity of the model gene (exons/isoforms) and confirm domains by HMM profiling (Figure 8).

**Figure 9:**
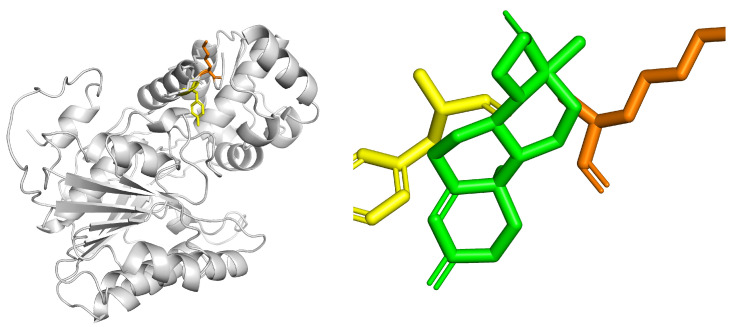
Three-dimensional structure of the VEP1/5β-reductase protein (**left**) from *Kalanchoe fedtschenkoi* modeled with AlphaFold. The overall conformation of the enzyme (gray) is shown with the catalytic motif Y381–K385 highlighted (Tyr381 in yellow and Lys385 in orange), characteristic of the SDR domain (short-chain dehydrogenase/reductase), and molecular coupling of progesterone (green) at the active site of the VEP1/5β-reductase protein (**right**).

Docking with progesterone showed an affinity of −6.8 kcal/mol, with the ligand carbonyl oriented towards Tyr381-OH (2.9 Å) and Lys385-NZ (3.1 Å) (Figure 9). Although the model lacks the NADPH cofactor, the observed geometry is consistent with the steroid reduction described in VEP1 of other plants.

The progesterone ligand (green) is oriented towards the catalytic motif Tyr381–Lys385 (yellow and orange, respectively). The interaction occurs in a hydrophobic cavity adjacent to the catalytic pair, with an estimated binding energy of −6.0 kcal/mol, suggesting moderate affinity and a possible arrangement compatible with the enzyme’s reductase function.

Molecular docking analysis between the VEP1/POR enzyme (5β-reductase) and the progesterone ligand (PROG) reveals a stable configuration within the hydrophobic channel characteristic of this oxidoreductase. Interaction distances were mainly between 2.8 Å and 4.0 Å, indicating a precise fit between the steroid substrate and the residues of the catalytic environment.

Among the closest residues are HIS A:186, ARG A:188, SER A:187, MET A:220, TYR A:226, and PHE A:227, which form a mixed network of hydrophobic contacts and weak hydrogen bonds that stabilize the steroid ring of PROG. The spatial arrangement suggests anchoring oriented toward the C3 carbonyl group of the steroid, consistent with the VEP1-catalyzed reduction mechanism in C-19 and C-21 steroids.

In Figure 6, the central circle represents the ligand (PROG) surrounded by residues of the catalytic pocket (green circles). The dashed green lines indicate hydrogen bonds (≤3.5 Å), and the dashed gray lines show van der Waals/hydrophobic contacts (≤4.2 Å). The solid border of the central circle distinguishes the main ligand from the residues. The distances (Å) are noted above each line.

The overall pattern confirms the structural complementarity of the VEP1 active site with progesterone-like compounds, reinforcing its role in modifying steroidal intermediates involved in the biosynthesis of brassinosteroids and bufadienolides in *Kalanchoe fedtschenkoi*.

## 4. Discussion

In the genus *Kalanchoe*, the main biological and pharmaceutical properties described in the literature include chemopreventive, antibacterial, anti-inflammatory, antiviral, antioxidant, and cytotoxic activities [5,36,82,83,84].

Comparing Table 1, Table 2 and Table 3, it can be observed that most experimental studies focus on antioxidant, antimicrobial, and anti-inflammatory activities, while clinical trials have primarily targeted applications related to pregnancy and the urinary system, especially in species of the subgenus *Bryophyllum* used in anthroposophic medicine.

The predominance of clinical studies conducted in Switzerland may be attributed to the active role of Swiss research groups in the clinical development of *Bryophyllum pinnatum.* These groups have historically investigated its use in gynecology and obstetrics, which may explain why most available clinical data originate from this region. Nevertheless, this geographic concentration represents a limitation, as further studies from other regions would enhance the generalizability of the findings. A limitation of the current evidence base is that most clinical trials have been performed in a single country, which may restrict extrapolation to other populations or healthcare contexts.

Despite the large number of identified compounds, many metabolites lack a detailed description of their bioactivity or molecular interactions. In their 2017 review, Kolodziejczyk-Czepas and Stochmal [85] compiled 31 bufadienolides from the genus *Kalanchoe*, although not all had demonstrated pharmacological activity. This metabolic diversity, characteristic of the group, represents both a strength and a challenge, as it makes it difficult to precisely assign functions and mechanisms of action to each metabolite.

Furthermore, regulatory, ecological, and legal aspects limit the progress of research and biotechnological applications. Some species are classified as invasive in various countries [86,87], which restricts their propagation and experimental use. In addition, increasingly stringent regulations for clinical trials [88,89] have reduced the number of authorized studies in Europe and other territories. Although patent production remains active, it depends on—and sometimes limits—experimental and clinical research [90,91].

The structural and in silico docking analysis performed in this work supports the finding that *Kalanchoe fedtschenkoi* possesses structural homologs of the enzymes CHS, CYP90, and VEP1, equivalent to those described in *Arabidopsis thaliana* and Medicago sativa [92]; however, functional activity cannot be inferred without biochemical validation. These enzymes support metabolic pathways associated with flavonoids, brassinosteroids, and bufadienolides, three groups of compounds with documented pharmacological relevance in the genus.

The chalcone synthase (CHS) conserves the catalytic triad Cys–His–Asn and exhibits affinity for naringenin chalcone, confirming its role in the condensation of p-coumaroyl-CoA with malonyl-CoA. The major flavonoids reported in *Kalanchoe* (e.g., quercetin, kaempferol, and their glycosides) are consistent with CHS-mediated biosynthetic flux and with the described antioxidant and anti-inflammatory properties [93,94]. The agreement between the docked poses and the orientations observed in 1CGK and 2CPP supports the reliability of the AlphaFold model for CHS. The conservation of the entry path and alignment with catalytic residues indicates that the predicted pocket geometry is compatible with productive substrate binding.

Clinically, standardized *Bryophyllum* extracts have shown sedative and tocolytic effects [95], suggesting a favorable safety margin for formulations rich in phenols and flavonoids, although the contribution of individual metabolites has not yet been isolated.

The CYP90 model showed conservation of the EXXR, PERF, and FxxGxxxCxG structural motifs, as well as stable coupling with campestanol oriented toward the heme group [96], consistent with typical CYP90A1/CPD-catalyzed oxidations in the brassinosteroid pathway [97,98]. This functional conservation suggests that *Kalanchoe* possesses the capacity to modulate steroid pathways involved in growth, resilience, and defense. In biomedical contexts, this metabolic axis is associated with bufadienolide and brassinosteroid profiles exhibiting selective cytotoxic activity and cytoprotective effects [99].

VEP1 (5β-reductase) exhibited the YxxxK motif characteristic of the SDR/PRISE family, with a hydrophobic cavity compatible with progesterone, suggesting a role in 5β-reductase. In *Arabidopsis thaliana*, its ortholog (StR1/VEP1) catalyzes similar reactions during the formation of iridoids and cardenolides [100]. In *Kalanchoe*, a functional VEP1 could facilitate the formation of 5β-reduced nuclei compatible with bufadienolides such as bryophyllins or bersaldegenins, compounds with recognized antitumor and antiproliferative activity [101].

The integration of in silico, experimental, and technological evidence allows us to delineate a coherent pattern: a flavonoid pathway (CHS), consistent with the antioxidant, anti-inflammatory, and wound-healing effects reported in in vitro and in vivo studies; a steroid and brassinosteroid pathway (CYP90), associated with hormonal regulation, stress defense, and cytoprotective activities; and finally, a bufadienolide pathway (VEP1), a plausible basis for the cytotoxic and antitumor properties widely documented in *Kalanchoe* and *Bryophyllum*.

Although bioinformatics approaches have contributed substantially to the identification of putative biosynthetic genes and to the theoretical exploration of the pharmacological potential of *Kalanchoe* metabolites, current evidence indicates that these predictions remain largely preliminary. Genome- and transcriptome-based analyses have begun to highlight candidate gene families involved in secondary metabolite biosynthesis, while docking and chemoinformatics tools have suggested possible targets and pharmacological activities. However, the validation of these predictions through experimental, clinical, or functional studies is still limited, underscoring the need for integrated multi-omics and mechanistic approaches to consolidate the bioactivity profiles proposed in silico. Bioinformatic insights are preliminary by nature and must be substantiated through robust preclinical and clinical studies.

Because no enzymatic assays were performed, the functional capacity of these predicted homologs remains unverified. The structural and docking results indicate compatibility with known active-site geometries but do not establish catalytic activity. Even so, significant knowledge gaps remain.

Although powerful approaches such as GNPS-based molecular networking, metabolomics–genomics integration pipelines, and biosynthetic gene cluster prediction platforms now allow direct connections between metabolites and their underlying enzymatic machinery, these strategies have rarely been applied in *Kalanchoe* or related Crassulaceae species. As a result, metabolite quantification in this group remains largely descriptive rather than mechanistically linked to specific genes or pathways.

Furthermore, clinical trials rarely use standardized extracts or define their precise chemical composition, which limits the reproducibility and pharmacological extrapolation of the results.

## 5. Conclusions

*Kalanchoe* is an important genus that needs to be thoroughly studied. The diversity in its production of metabolites is an important area of research, and more focused studies are required to clarify the specific action of each metabolite.

Despite being potentially invasive plants, *Kalanchoe* spp. contain metabolites of biological and pharmacological importance which can help in the development of new treatments for various ailments and diseases; however, worldwide regulations may limit the development of new studies and clinical trials.

Clinical trials are an important step in the development of new treatments for many diseases; however, laboratory research is the base of the knowledge needed for trials to be applied. Hence, more research in *Kalanchoe* metabolomics is needed to support the evidence of the effects of various metabolites.

Patents are an important part of the research process, and publication of different patents related to the metabolomics of the *Kalanchoe* genus can help to support clinical trials in the long term.

*The Kalanchoe* genus, including the *Bryophyllum* section, represents a diverse field in metabolomics research. Although there are various studies that support the efficacy of some *Kalanchoe* metabolites, their dynamics and relationship with other compounds, as well as the metabolization and disposal of these compounds, form an essential part in the development of new technologies and studies.

Further integrative multi-omics studies (genomics, transcriptomics, and metabolomics) are needed to trace the biosynthetic flow from gene to active metabolite. Functional assays of CHS, CYP90, and VEP1, along with quantitative LC-MS analyses and enzyme kinetics, could validate the catalytic predictions obtained through structural modeling and molecular docking.

Furthermore, expanding in silico studies using molecular dynamics and virtual ligand screening will allow for the exploration of synergistic interactions between compounds. From an applied perspective, harmonizing regulatory frameworks and creating open plant metabolomics databases would facilitate the safe and ethical clinical evaluation of *Kalanchoe* extracts and their derivatives.

The integration of literary, clinical, technological, and bioinformatics evidence demonstrates that species of the genus *Kalanchoe* possess the necessary molecular machinery to synthesize metabolites of high biomedical value. Structural and molecular docking analyses of CHS, CYP90, and VEP1 support the enzymatic plausibility of the flavonoid, brassinosteroid, and bufadienolide pathways, three metabolic axes directly associated with the therapeutic properties attributed to the genus.

This “evidence + mechanism” approach strengthens the link between clinical data and molecular principles and guides future biochemical validations, extract standardization, and the rational design of phytopharmaceutical formulations. Overall, *Kalanchoe* emerges as a promising but still underexplored model, capable of yielding new molecules with pharmacological potential through the integration of biotechnology, plant pharmacology, and structural bioinformatics.

We consider that the increasing use of bioinformatics approaches represents a rapidly expanding research avenue that has significantly accelerated multi-omics analyses by providing preliminary hypothesis-driven frameworks prior to experimental validation. These tools reduce exploratory uncertainty and help researchers avoid unsystematic or unguided experimentation. In this context, in silico analyses constitute a fundamental component of modern phytochemical and pharmacological research, as they not only guide the direction of experimental studies but also enable the integration of genomic, transcriptomic, metabolomic, and structural information. However, due to the intrinsic characteristics of bioinformatics tools—such as reliance on predictive algorithms, incomplete reference databases, variable annotation accuracy, and model-dependent assumptions—experimental validation remains essential to corroborate computational predictions. Therefore, while bioinformatics provides powerful insight into potential biosynthetic pathways, gene functions, and metabolite–target interactions, its outputs must be substantiated through rigorous empirical studies to confirm their biological and pharmacological relevance.

## Figures and Tables

**Table 1:** Studies on the *Kalanchoe* genus related to its biological or pharmacological activity.

Specie	Extract/Compound	Property	Organism	Reference
*K. pinnata*	Leaf juice concentrate	Hepatoprotective activity	Wistar rats	[33]
*K. pinnata*	Kaempferol and derivates	Antileishmanial activity	*Leishmania amazonenis* amastigotes	[34]
*K. daigremontiana*	Bufadienolides	Insecticidal activity	*Bombyx mori* larvae	[11]
*K. pinnata*	Bufadienolides	Anti-tumor-promoting activity	Raji cells	[10]
*K. daigremontiana*	Bufadienolides	Anti-tumor-promoting activity	Raji cells	[11]
*K. pinnata*	Aqueous leaf extract	Nephroprotective and antioxidant activity	Wistar rats	[35]
*K. daigremontiana*	Crude leaf extract	Hepatoprotective activity in diabetes	Wistar rats	[36]
*K. crenata*	Leaf extract	Anti-inflammatory and anti-arthritic activity	Wistar rats	[37]
*K. brasiliensis*	Aqueous leaf extract	Local anti-inflammatory activity	Swiss albino mice	[38]
*K. pinnata*	Aqueous leaf extract	Local anti-inflammatory activity	Swiss albino mice	[38]
*K. gracilis*	Methanolic stem extract	Antioxidant, anti-inflammatory, and antiproliferative activities	Murine macrophage cell line RAW264.7 and HepG2	[39]
*K. gracilis*	Methanolic stem extract	Analgesic and anti-inflammatory activities	ICR mice	[40]
*K. pinnata*	Bryophilline A and C	Insecticidal activity against third-instar larvae	Silkworm (Bombyx mori)	[41]
*K. pinnata*	KPB-100 and KPB-200	Virus inhibitors	HHV-2 and VACV	[42]
*K. prolifera*	Kaempferol and quercetin derivates	Cytotoxic activity	P-388 murine leukemia cells	[43]
*K. daigremontiana*	11α,19-dihydroxytelocinobufagin, bersaldegenin-1-acetate, and other bersaldegenin derivates	Antioxidant activity	Blood plasma	[44]
*K. tubiflora*	(6S,7R,8R,9S)-6-oxaspiro-7,8-dihydroxymegastigman-4-en-3-one	Anti-inflammatory activities	Murine macrophage cell line RAW264.7	[45]
*K. beharensis*	Methanol extract of K. beharensis	Insecticidal activity	*Spodoptera littoralis*	[46]
*K. longiflora*	Methanol extract of K. longiflora	Insecticidal activity	*Spodoptera littoralis*	[46]
*K. pinnata*	Aqueous extract	Antihypertensive activities	High-salt-loaded rats (SHR)	[47]
*K. fedtschenkoi*	Quercetin and caffeic acid	Antibacterial activity	ESKAPE pathogens	[5]
*K. mortagei*	Quercetin and caffeic acid	Antibacterial activity	ESKAPE pathogens	[5]
*K. pinnata*	Steam distillate of leaves	Antidiabetic activity	Streptozotocin-induced diabetic rats	[48]
*K. pinnata*	Quercetin, gallic acid, and quercitrin	Antiviral activity	Huh7it-1 cells	[49]
*K. gracilis*	Quercetin, gallic acid, and quercitrin	Antiviral activity	Enterovirus 71 (EV71) and coxsackievirus A16 (CVA16)	[50]
*B. pinnatum*	Ethylacetate fraction of the partitioned methanolic extract	Antidiabetic activity	Alloxan-induced diabetic rats	[51]
*K. pinnata*	Methanolic extract of roots	Antibacterial activity	*Escherichia coli Staphylococcus aureus, Pseudomonas aeruginosa*	[52]
*K. pinnata*	Aqueous extract and quercitrin	Antiallergic activity	Male BALB/c mice	[53]
*K. brasiliensis*	3,6-diamino-4,5-dihydroxyoctanedioic acid	Anti-inflammatory activity	Male C57B110 mice	[54]
*K. daigremontiana*	Kaempferol and derivates	Antiviral activity	Acyclovir-sensitive strains of HSV-1 and HSV-2	[55]
*K. daigremontiana*	Dichloromethane fraction of the ethanol extract	Cytotoxic activity	HeLa, SKOV-3, MCF-7, A375 cell lines	[56]
*K. blossfeldiana*	Ethanolic extract of leaves	Cytotoxic activity	HeLa cell line	[57]
*K. blossfeldiana*	Methanol extract	Antimicrobial activity	Diverse pathogenic bacteria, such as *Staphylococcus aureus and Escherichia coli,* among others	[58]
*K. pinnata*	Aqueous extract	Antinociceptive, antiedematogenic, and anti-inflammatory activities	Male Swiss mice	[59]
*K. gastonis-bonnieri*	Aqueous extract	Cytotoxic activity	Stromal cells from primary benign prostatic hyperplasia	[60]
*B. pinnatum*	Chewable tablets (100 mg dried BP matter in 1 g)	sedative and spasmolytic activity	Patients with restless leg syndrome	[61]
*K. flammea*	F82-P2 fraction of the extract, rich in coumaric acid and palmitic acid	Cytotoxic activity	PC-3 cells	[62]

**Table 2:** Clinical trials registered and extracted directly from the U.S. National Library of Medicine [63]. † symbol means a study has passed its completion date and its status has not been verified in more than 2 years.

Status	Study Title	Conditions	Interventions	Location
Not yet recruiting	Perceived Changes in Anxiety Symptom Burden During Treatment With *Bryophyllum* Pinnatum	Anxiety Symptoms	Drug: *Bryophyllum* 50%; chewing tablets	Unknown
Recruiting	Effectiveness of *Bryophyllum* in Nocturia-Therapy	Nocturia, Sleep Disorder	Drug: *Bryophyllum* pinnatum 50%; tablets into capsules (verum: 2 × 2 capsules/day)	University of Hospital, Clinic for Gynecology, Zurich, Switzerland
Completed	*Bryophyllum* Versus Placebo for Overactive Bladder	Overactive Bladder	Drug: *Bryophyllum* pinnatum; placebo in form of lactose	Department of Obstetrics and Gynecology, Zurich, Switzerland
Terminated	*Bryophyllum* Pinnatum Versus Solifenacin Versus Placebo for Overactive Bladder	Overactive Bladder, Urge Urinary Incontinence	Drug: *Bryophyllum*	Gynecologic Department, University Hospital Zurich, Zurich, Switzerland
Unknown †	*Bryophyllum* vs. Nifedipine	Tocolysis	Drug: *Bryophyllum* p.	Department of Obstetrics, University of Zurich, Zurich, Switzerland
Completed	The Impact of *Bryophyllum* on Preterm Delivery	Preterm Delivery, Preterm Contractions, Cervical Shortening	Drug: *Bryophyllum*; Other: Placebo	Obstetrical Unit, Women’s University Hospital Basel, Basel, Basel Stadt, Switzerland

**Table 3:** Patents in *Kalanchoe* genus associated with pharmacological or biological activities.

Plant Species	Patent	Patent Number	Reference
*Kalanchoe* sp.	Antioxidant composition	WO-2015002347-A1	[67]
*Kalanchoe pinnata*, *Kalanchoe daigremontiana*	Skin care composition	US-2020276256-A1	[68]
*Kalanchoe gastonis*	Cosmetic composition	KR-20140079896-A	[69]
*Kalanchoe* Gastonis-Bonnieri	Liquid composition for photodynamic therapy post treatment	KR-20150004092-A	[70]
*Kalanchoe flammea*	Extract with ethyl acetate for the treatment of prostate cancer	MX-2014015323-A	[71]
*Kalanchoe pinnata*	Method for antimicrobial peptide production	RU-2632116-C1	[72]
*Kalanchoe linearifolia*	Dermatological composition	EP-1857099-A1	[73]
*Kalanchoe brasilensis*	Cosmetic composition	BR-102015032217-A2	[74]
*Kalanchoe linearifolia*	Topical composition	FR-2900821-A1	[75]
*Kalanchoe* Gastonis-Bonnieri	Manufacturing method for antioxidant composition	KR-20140142531-A	[76]
*Kalanchoe pinnata*	Composition for skin care and protection	FR-3000390-A1	[77]
*Kalanchoe* Gastonis-Bonnieri	Composition using iontophoresis	KR-20150047040-A	[78]
*Bryophyllum* sp.	Medicinal preparation	CN-104107209-A	[79]
*Bryophyllum pinnatum*	Preparation of Chinese herbal recipe	US-2005158402-A1	[80]
*Bryophyllum pinnatum*	Herbal composition for the treatment of burns	WO-2020201847-A1	[81]

## Data Availability

The data presented in this study are openly accessible in the NCBI GenBank repository under reference number GCA_002312845.1 (v1.1) and are associated with the publication DOI: 10.1038/s41467-017-01491-7.

## Supplementary Materials

The following supporting information can be downloaded at: https://www.mdpi.com/article/10.3390/biotech14040097/s1, Figure S1: CHS model structural validation; Figure S2: CYP90 model structural validation; Figure S3: VEP1 (POR) model structural validation.
