# Supplementary material for: From Traditional Use to Molecular Mechanisms: A Bioinformatic and Pharmacological Review of the Genus Kalanchoe with In Silico Evidence
Source: BioTech (Basel). 2025 Dec 12;14(4):97. doi: 10.3390/biotech14040097 (PMC12730917; doi:10.3390/biotech14040097)
Supplement: Supplementary file 1 [file biotech-14-00097-s001.zip › biotech-4028662-supplementary.pdf]

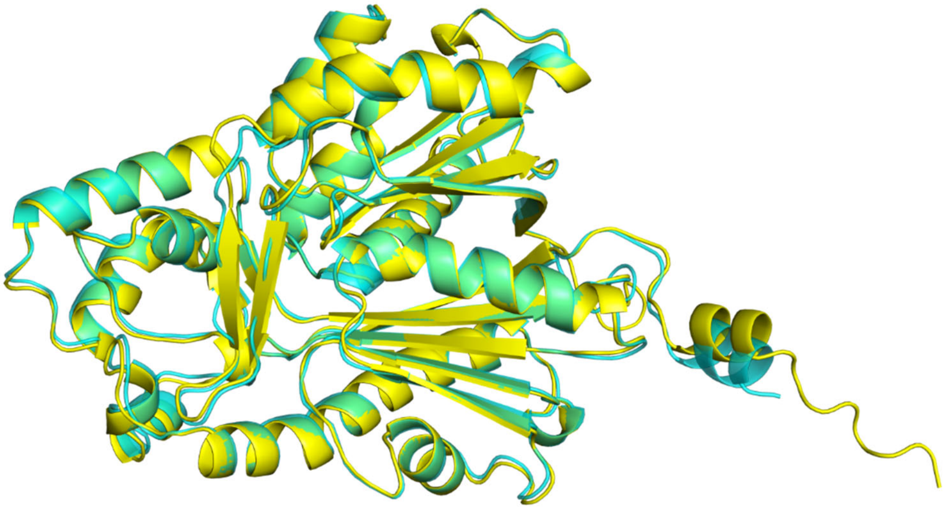

Supplementary Figure S1 – CHS model structural validation. Superposition of the K. *fedtschenkoi* AlphaFold-predicted CHS structure (yellow) with the crystallographic chalcone synthase template 1CGK (cyan). The alignment reveals a high degree of conservation across the canonical type III polyketide synthase (PKS) β-trefoil fold, including the characteristic arrangement of alternating α-helices and β-strands that form the catalytic architecture. Core elements of the active site cavity—such as the positions of the catalytic triad residues (Cys-His-Asn) and the geometry of the substrate-binding tunnel—align closely between both structures, supporting the structural reliability of the predicted model. Minor deviations are observed primarily in flexible loop regions, which are known to vary across CHS homologs and are not expected to significantly alter enzymatic function. Overall, the overlay supports the accuracy of the AlphaFold model for downstream docking and functional inference.

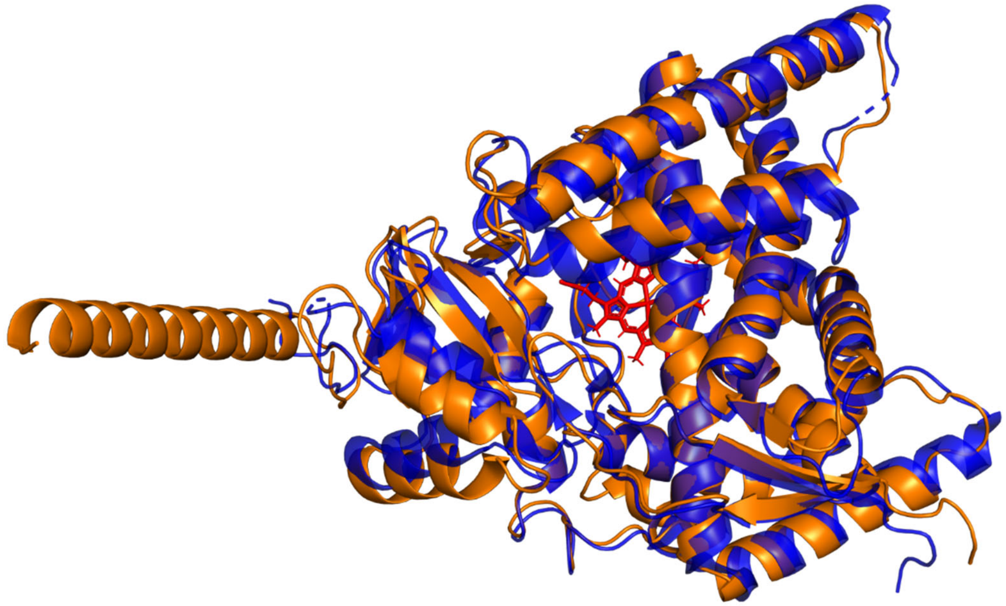

Supplementary Figure S2 – CYP90 model structural validation. Structural superposition of the AlphaFold-predicted CYP90 model (orange) with the experimentally resolved Arabidopsis thaliana CYP90B1 structure 6A15 (blue). The alignment demonstrates strong conservation of the canonical cytochrome P450 fold, including the arrangement of the helices forming the I-helix, the meander region, and the β-sheet domain characteristic of plant P450s. Importantly, the orientation of the prosthetic heme group (red) and the geometry of the heme-binding pocket—including the conserved cysteine axial ligand—are preserved between both structures. This conservation suggests that the AlphaFold model maintains a catalytically competent architecture. Minor deviations are restricted to peripheral loops and the N-terminal region, which commonly display flexibility in membrane-associated P450s. Overall, the structural alignment supports the reliability of the CYP90 model for downstream active-site analysis, ligand docking, and functional interpretation.

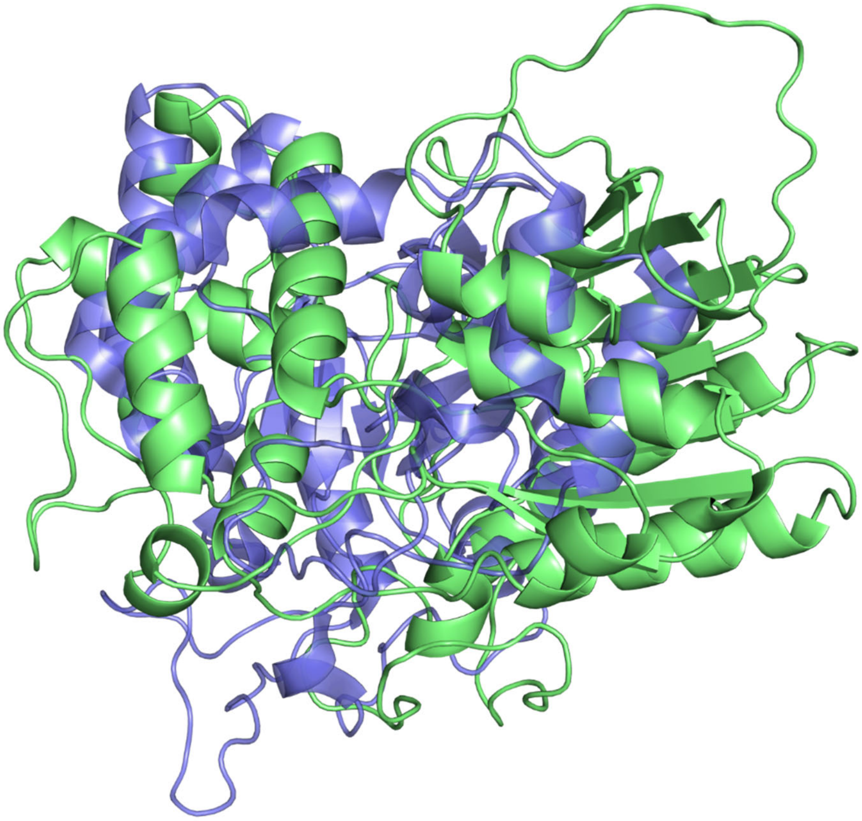

Supplementary Figure S3 – VEP1 (POR) model structural validation. Structural overlay between the AlphaFold VEP1 model (lime) and the aldo-keto reductase template 4JIR (slate). Although both proteins share the canonical aldo-keto reductase / short-chain dehydrogenase–reductase (AKR/SDR) fold—characterized by a central β-sheet flanked by α-helices—the superposition shows greater divergence relative to the CHS and CYP90 alignments. This reduced structural overlap is expected for AKR/SDR enzymes, which constitute a highly versatile superfamily with substantial variability in substrate-binding loops, cofactor-interaction regions, and terminal extensions. The most pronounced differences arise in flexible loop regions and the C-terminal tail, which are known to adopt species-specific conformations. Despite these peripheral deviations, the conserved catalytic tetrad and the core β-α-β architecture remain aligned, supporting the functional plausibility of the model for mechanistic interpretation and docking analyses.
